# Recurrent Bacterial Meningitis in an Infant Caused by a Sacral Dermal Sinus Tract

**DOI:** 10.7759/cureus.107220

**Published:** 2026-04-17

**Authors:** Samuel Stresemann, Mollie Westrick, Taylor Fitch, Omayma Amin

**Affiliations:** 1 Department of Pediatrics, Emory University School of Medicine, Atlanta, USA

**Keywords:** bacterial meningitis, congenital spinal anomaly, fever of unknown origin, fever without a source, intradural cyst, magnetic resonance imaging, recurrent meningitis, sacral dermal sinus tract, spinal dysraphism

## Abstract

Congenital dermal sinus tract is a rare form of closed neural tube defect, connecting the epidermis to a deeper layer. It presents in only one in 2,500 live births. This connection between the outside world and the spinal cord is a nidus for bacterial meningitis and other infections, and dedicated imaging is essential in identifying such a tract. We present a case of a 9-month-old previously healthy male who repeatedly presented with intermittent fevers despite recurrent antibiotics. Cerebrospinal fluid (CSF) analysis consistently demonstrated neutrophilic pleocytosis, elevated protein, and hypoglycorrhachia, while extensive testing for autoimmune etiologies of fever was normal. Whole-body magnetic resonance imaging (MRI) and head computed tomography were negative. He developed an altered mental status, and CSF continued to suggest bacterial meningitis; CSF cultures grew *Propionibacterium acnes*. Imaging showed hydrocephalus, and an external ventricular drain was placed. A dedicated MRI of the brain and spine was obtained, revealing debris in the lateral ventricles, ventriculitis, leptomeningitis, and a sacral dermal sinus tract with an associated intradural cyst extending from L5-S4. This finding was correlated with a small midline sacral dimple. The patient underwent correction of the dermal sinus tract and removal of the cyst. He improved with ceftriaxone and metronidazole along with placement of a ventriculoperitoneal shunt. This case underscores the importance of a thorough and repeatedly performed physical exam in infants with a history of recurrent fever without a known source. In a patient with recurrent bacterial meningitis, the cause is likely anatomical, and detailed imaging, notably a dedicated spine MRI in this case, is indicated to fully investigate the source even when whole-body MRI demonstrates no abnormal findings.

## Introduction

Neural tube defects result from abnormal formation of the neural tube during embryonic development, and are one of the most common types of congenital defects [1]. They are thought to occur between three and eight weeks of gestation as the ectoderm is differentiating into neural and cutaneous counterparts [2,3]. Closed neural tube defects occur when the spinal cord forms incorrectly, but the skin overlying the defect is closed. These defects can present with cutaneous skin findings of dimples or other lesions on the back [1].

Congenital dermal sinus tract is a rare form of closed neural tube defect, with an overall incidence of one in 2,500 live births, occurring when a tract remains that connects the epidermis to a deeper layer, such as the spinal cord [2-4]. This sinus tract can occur alongside other pathologies, such as a tethered cord and intradural mass [3]. The connection between the skin and the outside world with the spinal cord is a nidus for central nervous system (CNS) infection [5]. Specifically, it can result in recurrent bacterial meningitis, defined as two or more episodes of meningitis caused by different bacterial organisms or two episodes caused by the same organism with a period of recovery in between [6]. Recurrent bacterial meningitis itself is a rare phenomenon, but it is most commonly due to anatomic variants; across all age groups, dermal sinus tracts and dermoid cysts represented approximately 7% of the total cases [6]. Thus, patients with episodes of recurrent meningitis require further workup for potential anatomic anomalies. In this case report, we describe a 9-month-old infant with recurrent bacterial meningitis caused by a sacral dermal sinus tract, which was not identified despite multiple provider evaluations and whole-body magnetic resonance imaging (MRI), requiring a dedicated spinal MRI to establish the diagnosis.

## Case presentation

A 9-month-old previously healthy male presented to an outside hospital for fever and was found to have acute otitis media (AOM). He was admitted for observation due to one episode of supraventricular tachycardia (SVT). At this time, laboratory studies showed a high-normal white blood cell count, an elevated absolute neutrophil count, and thrombocytosis (Table 1). He was treated with ceftriaxone, given two prior episodes of AOM, which had been treated with amoxicillin and amoxicillin-clavulanic acid, respectively. Blood cultures were negative. He had no further recurrence of SVT, and with his fever now resolved, he was discharged from the hospital.

**Table 1:** Serum and CSF studies at initial admission, fifth admission, and sixth admission for fever Bold values indicate results outside the reference range. "—" indicates test not performed.

Test	Reference Range	Initial Admission	Fifth Admission (1 Month Later)	Sixth Admission (2 Months Later)
Serum Studies				
White blood cell count (×10³/µL)	6.0–17.0	15.3	26.0	32.0
Absolute neutrophil count (×10³/µL)	0.96–8.33	9.6	23.0	25.5
Platelet count (×10³/µL)	150–450	648	509	729
Cerebrospinal fluid studies				
CSF white blood cells (cells/µL)	0–30	—	380	178
CSF protein (mg/dL)	12–36	—	180	100
CSF glucose (mg/dL)	40–70	—	15	35

**Figure 1:**
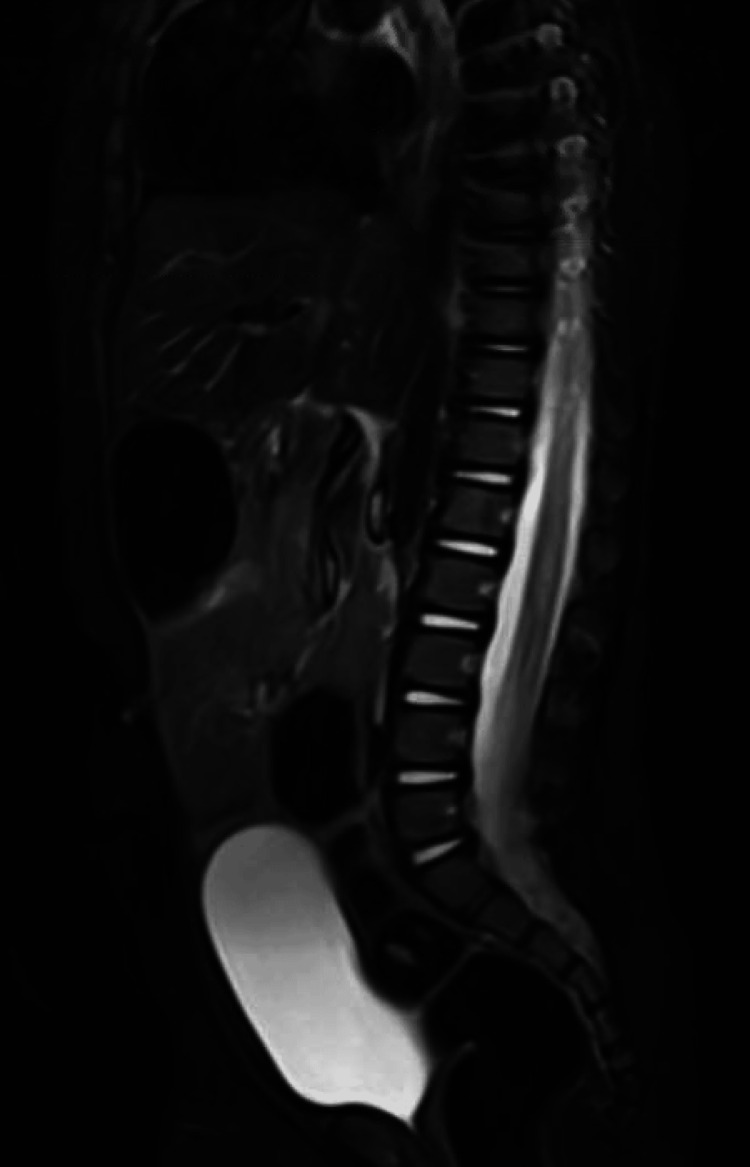
Spinal section of initial whole-body T2-weighted short tau inversion recovery MRI (sagittal view) demonstrating no abnormal findings With no abnormalities seen at the L5–S4 levels, this highlights the limitation of whole-body MRI in detecting subtle spinal pathology such as a dermal sinus tract.

Over the course of the next three weeks, he repeatedly presented to the hospital for fevers on three more separate occasions. He was primarily admitted for persistent fevers during these hospitalizations, but he was also noted to have several episodes of emesis on his fourth admission. Throughout these hospitalizations, his imaging workup consisted of abdominal ultrasound, echocardiogram, chest X-ray, computed tomography of the abdomen and pelvis with contrast, and whole-body MRI which were all normal (Figure 1). Other than his first admission for SVT, all subsequent electrocardiograms were normal.

One month after his first admission, he presented again for a fifth time with fever and was also found to be lethargic, although arousable. Neurological exam revealed no other abnormalities. He had significant leukocytosis compared to a month prior, elevated absolute neutrophil count, and thrombocytosis. Analysis of his cerebrospinal fluid (CSF) demonstrated neutrophilic pleocytosis, elevated protein, and low glucose (Table 1). Culture and Gram stain of CSF grew a colony of Propionibacterium acnes. Meningitis/Encephalitis polymerase chain reaction (PCR) on this admission was negative. He was found to have hydrocephalus, had a ventriculostomy and external ventricular drain (EVD) placed, and was started on a 10-day course of ceftriaxone, after which further CSF cultures were negative. With the EVD placement, his mental status improved back to baseline, and the EVD was removed five days later. He was noted to have pneumatosis, which was treated medically with metronidazole. Due to the recurrence of the fever without a clear etiology, workup for autoimmune and immune dysregulation was begun. Workup, including cytokine panels, immunoglobulin G levels, neopterin CSF level, C-X-C motif chemokine ligand 9 levels, antinuclear antibody, and autoimmune encephalitis antibodies, was unremarkable.

**Figure 2:**
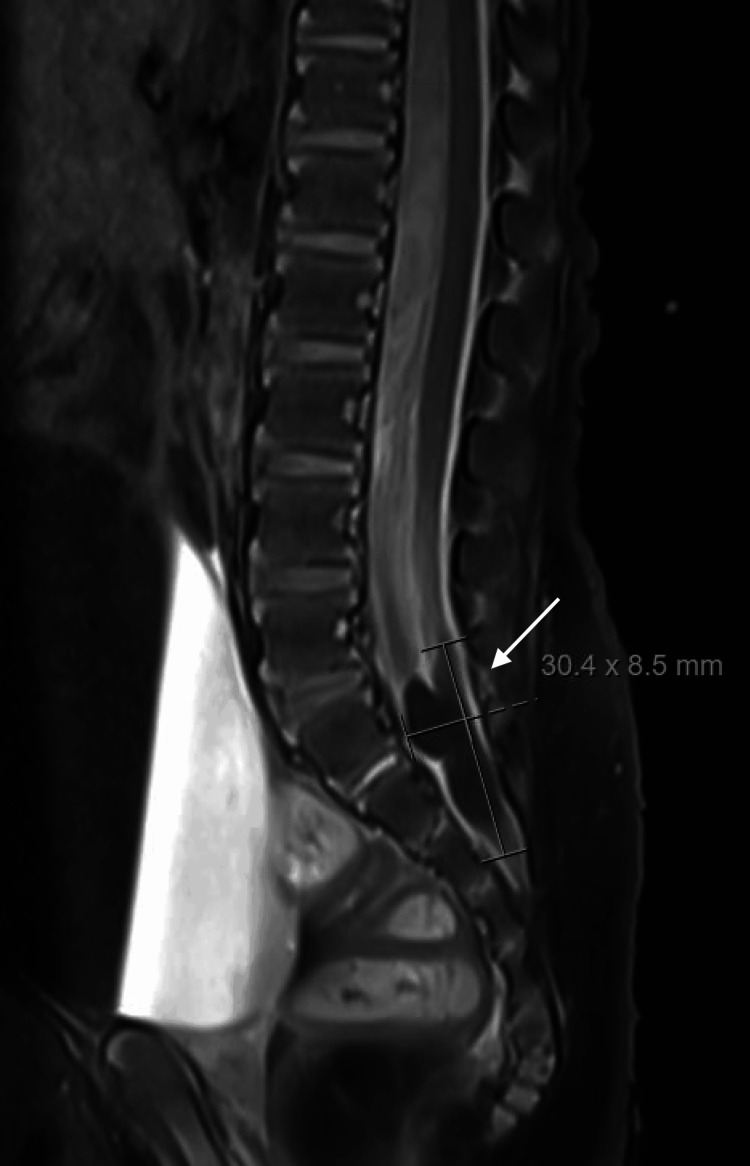
T1-weighted turbo spin-echo fat suppression spinal MRI (sagittal view) demonstrating an intradural cyst at the L5–S4 levels (white arrow) The intradural cyst was only detected on a dedicated spine MRI, highlighting the need for specific imaging to identify this anatomic anomaly.

**Figure 3:**
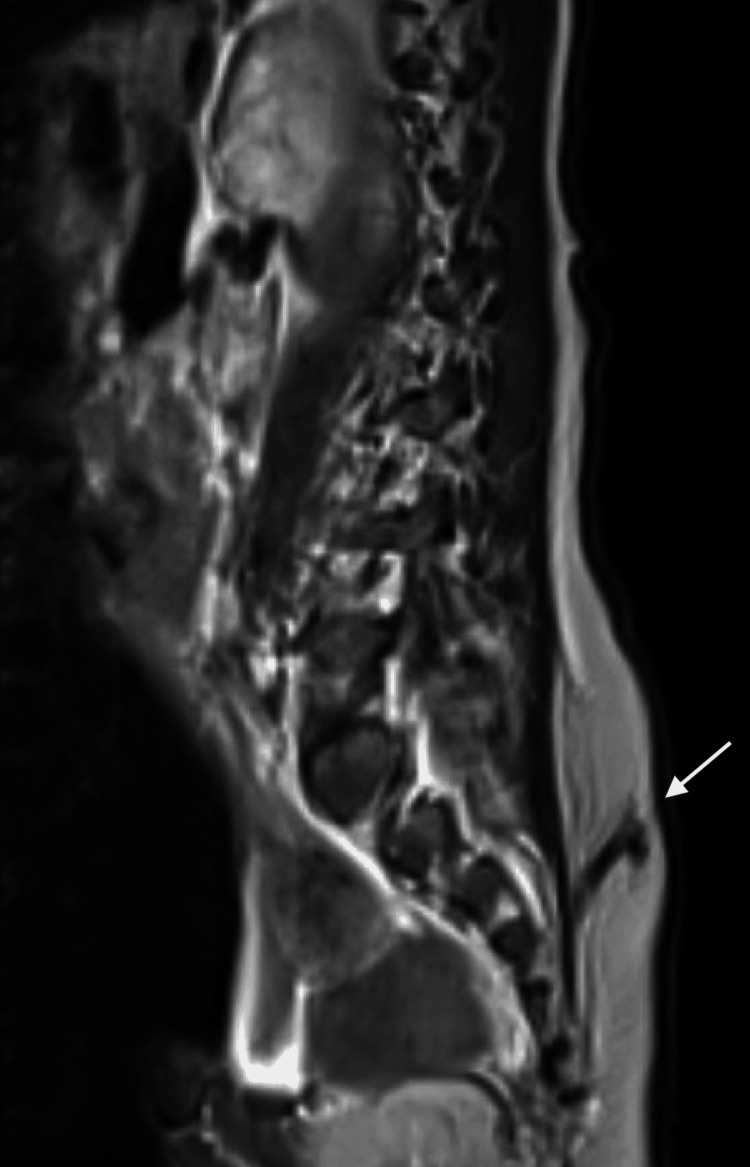
T1-weighted fluid-attenuated inversion recovery spinal MRI (sagittal view) demonstrating sacral dermal sinus tract (white arrow) The sacral dermal sinus tract served as the definitive diagnostic finding to establish a communication between the skin and spinal canal, explaining the patient’s recurrent meningitis.

On his sixth admission for fever, he was transferred to our center for further workup of two months of recurrent fever without a known source. On arrival to our intensive care unit, similarly to his previous admission, he presented with leukocytosis, elevated absolute neutrophil count, and thrombocytosis. Repeat CSF analysis revealed neutrophilic pleocytosis, elevated protein, and low glucose (Table 1). CSF culture obtained four days after the last antibiotic dose grew no organisms. He was noted to have signs of increased intracranial pressure, warranting EVD replacement. A dedicated MRI brain and spine was obtained, revealing leptomeningitis, ventriculitis, and a sacral dermal sinus tract with intraspinal extension and a peripherally enhancing collection in the distal thecal sac extending from L5-S4 with findings of meningeal inflammation (Figures 2, 3). This finding was correlated with a small midline sacral dimple.

He was started on ceftriaxone and metronidazole with notable clinical improvement, followed by lumbosacral laminoplasty for excision of the dermal sinus tract and removal of an intradural cyst. Next-Generation Sequencing (NGS) of CSF resulted as Prevotella bivia, an anaerobic gram-negative bacillus. Whole exome sequencing resulted as non-diagnostic. Given improvement with EVD placement and irritability with EVD clamping, a ventriculoperitoneal shunt was placed. The patient made a full clinical recovery.

## Discussion

With improved diagnostics and treatments, our understanding of rare diseases is improving tremendously. However, we must not forget about more common diagnoses. Examining the lumbar and sacral spine is an essential part of the physical exam in neonates and infants. Moreover, the differential diagnosis for fever of unknown origin is vast, with the history and physical being the best clues to possible etiologies; the physical exam itself should be conducted multiple times even if it has been thoroughly done in prior evaluations [7]. Provider recognition of sacral dimples and signs of closed neural tube defects is essential in providing care to patients with such defects [1]. MRI remains the gold standard for workup when a sacral dimple is identified [4]. Early intervention on dermal sinus tracts and tethered cords may lead to improved outcomes later in life. Even with access to exceptional diagnostic testing, it is important to always conduct a thorough physical exam and investigate abnormal findings.

Hydrocephalus is one of the most common presentations in patients with tuberculous meningitis (TBM) and thus must be considered in a patient with chronic fever and hydrocephalus. Presence of hydrocephalus in those with TBM is associated with severe disease and adverse outcomes, and thus it is essential to conduct workup for TBM in patients for whom the diagnosis is yet unclear [8]. CSF in bacterial meningitis will typically show neutrophilic pleocytosis, elevated protein, and low glucose, compared to TBM, which usually results in a lymphocytic pleocytosis [9]. Congenital abnormalities represent a common cause for recurrent bacterial meningitis, which creates a pathway for bacteria to invade the CNS [6]. Because of this, patients with suspected recurrent bacterial meningitis should be worked up for potential causes, including a dedicated spine MRI [5].

Although cultures growing skin flora, including Propionibacterium species, are often thought to be contaminants rather than causes of infection, they must be considered as an etiology of illness in those with an indeterminate origin [10]. In addition to leukocytosis with left shift, thrombocytosis is associated with infection and thus should increase concern for infection [11]. From a clinical perspective, it is easier to disregard known contaminants as the cause for infection, especially with imaging results not demonstrating an internal connection to the skin. Since it is known that Propionibacterium acnes is a common etiology of post-neurosurgical meningitis, which facilitates a similar method of infection as a dermal sinus tract, it makes it more than likely that the Propionibacterium culture result was a true positive [12]. If this result had been investigated further instead of being ruled as a contaminant, it may have led to an earlier diagnosis of the sinus tract.

This case also highlights the utility of NGS, since bacterial meningitis remained on the differential although cultures and PCR were negative [13]. Antibiotic pretreatment decreases the yield of bacterial culture and PCR [14]. However, antibiotic treatment does not affect the validity of NGS. Prevotella bivia has been reported as the cause of meningitis in an infant with negative CSF cultures, only diagnostically discovered once NGS was performed [15].

Recent research into autoimmune encephalitis has increased awareness and diagnostic tools for this category of illness. Recurrent or prolonged symptoms should prompt investigation of autoimmune encephalitis, though MRI often shows hyperattenuation in the temporal lobe rather than findings of ventriculitis seen in this patient. Additionally, CSF findings in autoimmune encephalitis rarely demonstrate neutrophilic pleocytosis with white blood cell counts over 100, which was present in our patient. Furthermore, although there are reports of seronegative autoimmune encephalitis, this diagnosis should be made cautiously, given other causes of such symptoms. In this case, the patient’s immune workup was normal, making immune involvement less likely [16,17]. Although rare diseases and processes such as immune dysregulation should remain on the differential for fever of unknown origin, it is important to rule out more common etiologies. It can be tempting to go looking for rare causes of illness, but we must be aware of this bias and remember to look for horses and not zebras when we hear hoofbeats [18].

## Conclusions

This case emphasizes the role of the physical exam in infants, especially the examination of the spine. Recurrent CSF findings consistent with bacterial meningitis warrant investigation for a source, including a thorough physical exam and detailed imaging, especially a dedicated spine MRI, regardless of a previous, whole-body MRI. Even with access to such exceptional diagnostic testing, no test is perfect. When findings repeatedly point toward a specific disease process, clinicians should continue investigating the underlying source rather than looking for rarer alternatives.
